# Telehealth in breast cancer following the coronavirus disease 2019 pandemic

**DOI:** 10.37349/etat.2023.00195

**Published:** 2023-12-26

**Authors:** Jean Zeghondy, Elie Rassy, Pietro Lapidari, Roland Eid, Barbara Pistilli

**Affiliations:** University of Campania “Luigi Vanvitelli”, Italy; Nicola Normanno, Istituto Nazionale Tumori-IRCCS-Fondazione G. Pascale, Italy; Medical Oncology Department, Gustave Roussy, F-94805 Villejuif, France

**Keywords:** Telehealth, telemedicine, breast cancer, breast neoplasms, coronavirus disease 2019, pandemic

## Abstract

Breast cancer (BC) is the second most diagnosed cancer in 2018 with around 2.3 million cases globally in 2020. In March 2020 and after its worldwide spread, the World Health Organization (WHO) declared the coronavirus disease 2019 (COVID-19) outbreak, a respiratory disease caused by the severe acute respiratory syndrome coronavirus 2 (SARS-CoV-2) virus, a pandemic. During this time, cancer patients were heavily impacted and their treatment plans were changed due to measures to fight the disease and solutions had to be found to maintain their follow-up and management from a distance. Some cancer groups worldwide have recommended then the use of telemedicine for oncology patients to ensure the continuity of medical care during the pandemic. This method was considered effective and clinicians worldwide continued using telehealth even after the cessation of worldwide restrictions. To this end, current up-to-date data on the use of telemedicine in BC patient after the COVID-19 outbreak are summarized in this narrative review.

## Introduction

Worldwide, approximately 18.1 million new cancer cases were reported in 2020 [1]. Breast cancer (BC) is the second most diagnosed cancer in 2018 just behind lung cancer [2], with around 2.3 million cases globally in 2020 [1]. It is estimated that 1 in 8 women will be diagnosed with invasive BC during the course of their life and that 3% of all women will die from metastatic BC [3].

In terms of survival, between 2010 and 2014, the 5 years overall survival in Australia and the United States (US) was around 90 percent in contrast with India with 66.1 percent [4]. The global burden of disease in disability-adjusted life-years (DALYs) was estimated to be over 20 million in 2019, with a regressing trend in high social-development index (SDI) countries, in contrast to a rise in the trend in low SDI countries [5]. This disparity between countries and within countries and communities, as well as the high incidence of BC and the complexity of diagnosis and management makes BC a global public health concern [6]. In March 2021, the World Health Organization (WHO) launched the Global BC Initiative, in order to offer guidance on improving the management of BC, based on three pillars: health promotion and early detection; timely diagnosis; and comprehensive management [7].

The detection of BC at an early stage reduces BC related mortality. Indeed, women participating in regular mammography screening had a 41 percent reduction in mortality within 10 years, especially that there was a 25 percent reduction in the rate of advanced BC detection [8]. Worldwide, most guidelines recommend an annual or biennial mammography screening for women at average risk, between the ages of 50 years to 69 years (40–74 years) [9].

Since BC is a heterogeneous disease, its management depends on a variety of molecular characteristics in addition to the usual staging at diagnosis. Alongside testing for estrogen receptors (ERs), progesterone receptors (PRs), and human epidermal growth factor receptor 2 (HER2), testing for antigen Ki-67 usually allows a stratification of patients based on prognosis and the risk of recurrence [10]. Recently, genomic assays also joined the arsenal in treatment decision making [11]. Treatment possibilities include surgery, radiation therapy, chemotherapy, hormonal therapy and/or targeted therapy. The management of BC is therefore multidisciplinary and necessitates a good communication among the team of care and between the team of care and the patients [12].

Coronavirus disease 2019 (COVID-19) is a respiratory disease caused by the severe acute respiratory syndrome coronavirus 2 (SARS-CoV-2) virus. After its worldwide spread, it has been declared a pandemic by the WHO in March 2020 [13]. This novel viral infection was associated with many complications including acute respiratory distress syndrome (ARDS), sepsis and septic shock, heart failure, arrhythmias and hypercoagulability. These disease complications were mainly seen in patients with older age or patients with underlying medical comorbidities. Since cancer patients were considered at a higher risk of COVID-19 related complications, they were heavily impacted by the pandemic [14]. Moreover, for many patients, treatment plans were changed due to measures to fight the pandemic, and many reported distress and fear [15].

Different measures have been taken to reduce the infection rate, ranging from severe lockdowns to lighter social distancing measures and mask mandate. The Senologic International Society (SIS) published data showing that the majority of screening programs, magnetic resonance imaging and surgeries for patients with comorbidities, reconstructive goals and benign disease were halted during the pandemic [16]. In lights of restricted access to care, as well as prioritization of patients allowed at the clinics, a solution had to be found to bring the care team together as well as to allow follow-up and management of patients from a distance.

An ideal solution to connect patients to physicians without unnecessary exposure to infectious risk is the possibility to practice medicine without the necessity of physical presence. Telemedicine by definition is providing health care services, at a distance, using telecommunication services, for the benefit of the patients [17]. The first reported experiments date back to 1976 when telemedicine has been used to serve remote regions in Canada [18]. Technologies used in this context varied throughout the years from telephone calls, to emails and video conferences, with or without special equipment and applications.

The use of telemedicine for BC patients is nothing new. In fact, a study from 1995 reports the satisfaction of women in remote regions having access to support groups through audio-teleconferencing [19]. A 2000 paper, written by Olver and Selva-Nayagam [20], also reports the beneficial role of telemedicine in multidisciplinary team meetings between centers on both ends of Australia, from both patients and physicians’ perspectives, in reducing travel time and costs. The application of telemedicine in multidisciplinary meetings for BC has been reported in the TELEMAM trial that described a high concordance in decision making between physical and virtual meetings over a year. However, the authors reported the need for 40 meetings per year to make virtual meetings cheaper than in-person boards [21]. The use of mobile health applications was also shown to be positively affecting the sexual health outcomes of BC patients [22].

## Telemedicine and BC during the pandemic

During the COVID-19 pandemic that started in March 2020, some cancer groups worldwide have recommended telemedicine for oncology patients. The European Society of Medical Oncology (ESMO), published an expert consensus where the first statement considers digital health as a valuable tool for counselling, coordination, prescribing, and assessment among others [23]. The American Society of Clinical Oncology (ASCO), also discusses the role of tele oncology in clinical trial consent and enrollment as well as in palliative settings. It also reports the satisfaction of both sides: practitioners and patients [24]. Seventy-one percent of responders in an SIS study preferred telemedicine for BC patients when possible [16]. Its widespread use as well as its effectiveness led it to be called “The Youngest Pillar of Oncology” by Pareek et al. [25] in an article published in the *Journal of Clinical Oncology* in 2020. Additionally, a survey by a workgroup from the National Comprehensive Cancer Network (NCCN) of physicians treating oncologic patients, reports that in 93% of cases no or rare adverse events related to telemedicine occurred, and that 46% of post-pandemic visits can be virtual [26]. The rise of telemedicine during the pandemic was clearly reflected in Google search trends, where between September 2019 and September 2020, there was a 907.1% raise in search interest for telemedicine in contrast to 30.6% decrease in search interest for BC [27].

Patients with BC benefited from telemedicine during the pandemic. Indeed, in some cases it was possible to switch and follow-up, at a distance, patients with positive hormone receptors BC from neoadjuvant or adjuvant chemotherapy to hormonal therapy, or those with negative receptors BC to oral chemotherapy [25]. The ESMO recommended implementing safety monitoring through telemedicine for early stages BC as well as metastatic BC, where it also recommended switching to oral chemotherapy when possible [28]. As reported in an Italian study, telemedicine follow-up at a distance of patients discharged a day after mastectomy showed low post-operative complications rate. It also allowed to maintain the concerned institution without confirmed COVID-19 cases for a certain period of time [29]. These results were similar to a Spanish study where patients were satisfied with being assessed remotely before surgery and followed-up after that using teleoncology [30]. Positive results were also reported for drain and dressing tele management after breast reconstructive surgery [31].

A Turkish study reports that among the studied population, BC patients were those who benefited the most from telemedicine and that overall, 92.8% of their population needs were satisfied with telemedicine, specially that it avoided traveling through the countries, under strict lockdown rules [32]. The most used method in the study was audio calls, followed by video calls and text messages [32]. Another Italian study of satisfaction for patients with BC, shows that 80.3% of participants were satisfied with electronic medical record-assisted telephone follow-up (E-TFU) to minimize hospital exposure [33]. In the US, patients enrollment in BC clinical trials was drastically impaired at the beginning of the pandemic, however, using virtual visits as well as electronic signatures for consent allowed a better participations in these trials without unnecessary exposure to hospitals [34]. Experiences of the use of telemedicine in the frame of multidisciplinary BC meetings also show higher rates of participation, better decision-making and more convenience for participants [35].

A multicentric French and Italian study by Bizot et al. [36] with 1,299 participants with BC that benefited from telemedicine, mostly (90%) by telephone, reports a high satisfaction rate from patients, with a mean EORTC OUT-PATSAT 35 score of 77.4 [36]. Video calls in contrast to phone calls were significantly more satisfactory, and an advanced disease stage was also significantly associated with poorer satisfaction from telemedicine [36]. This study also reported a significantly lower satisfaction score for patients with anxiety in comparison with patients with no or low anxiety [36]. Moreover, the use of telemedicine during the pandemic also facilitated holistic care interventions, such as yoga therapy, which showed a reduction in anxiety, fatigue and distress in BC patients [37]. Examples of studies on telemedicine and BC during the pandemic are mentioned in Table 1.

**Table 1:** Examples of studies on telemedicine and BC during the pandemic

**Author, Year**	**Country**	**Study type**	**Population size**	**Tool**	**Goal**	**Main results**
Pelle et al. [29], 2020	Italy	Monocenter experience	79 Patients	Voice calls, messaging Apps	F/U of discharged patients after mastectomy	No COVID-19 infections No early or late surgical complications
Sharp and Masud [31], 2021	UK	Case report	1 Patient, post-operative	Emails, voice calls, photographs	COVID-19 monitoring, drain removal, wound care	Uneventful full recovery
Yildiz and Oksuzoglu [32], 2020	Turkey	Cross-sectional, descriptive	270 BC patients (total 421 patients)	Voice calls, video calls, messaging Apps	F/U	Postpone unnecessary in-person visits Referral to testing without physical visit
Merz et al. [33], 2021	Italy	Prospective survey	137 Patients	Voice calls E-TFU	F/U	Decrease unnecessary exposure, satisfaction of most patients
Ndumele and Park [34], 2021	US	Electronic search	N/A	Emails, voice calls	Enrollment in clinical trials	Possibility of electronic consent, prescriptions, [***]F/U[/***]

F/U: follow-up; N/A: not available

## Telemedicine and BC beyond COVID-19

While the strict restrictions during the COVID-19 outbreak justified for many the use of telemedicine in oncology, leading to a peak in its usage, whether it will be used in the aftermath or not is not certain. The chances of telemedicine to survive in post-pandemic settings depend on its capacity to overcome technical problems as well as barriers related to the need of human contact for patients that suffer from distress [38].

A Brazilian group published a protocol of a randomized control trial where they compare cancer-related fatigue and quality of life in two groups of patients with non-metastatic BC. While the first group will receive twice a week a multicomponent training program, including aerobic, balance and resistance home-based exercises supervised by video calls, in addition to multicomponent training and health education (MTHE) group, the second group will benefit from a once-a-week session of health education (HE) alone, where medical information will be shared by text and then discussed with a small group. Their research hypothesis is that MTHE is superior to HE alone for all of the studied criteria [39]. Another Brazilian group published a study protocol on the role of telenursing in sexual function for patients undergoing BC treatment, where they intend to compare the Female Sexual Function Index (FSFI) between two groups of patients, before, at the end and 12 weeks after the intervention. The first group will receive standard care with no extra intervention while the second group will receive three sexual education sessions through telephone calls for a period of six weeks [40].

Gordon and colleagues [41] published a study protocol where they use a smart speaker as a support tool for their nurse Addressing Metastatic Individuals Everyday (AMIE) virtual assistant. This assistant assesses symptoms and gives nutritional advice. They intend to compare metastatic BC patients using Nurse AMIE with a smart speaker to those using AMIE over a three-month period. The primary outcome was change in physical distress over three months, and secondary outcomes included feasibility, acceptability, patient reported outcomes [41]. Smart speakers are voice-based artificial intelligence devices, and help increasing access to telemedicine, especially for patients who feel overwhelmed by technology. A qualitative study with Nigerian BC patients showed that patients valued the input of telehealth in terms of HE on chemotherapy, exercise and diet, as well as psychological support. Participants expect that the use of technology can answer these unmet needs in a developing country [42].

The feasibility of pelvic floor training through video call for women with BC has also been studied, showing significantly positive results in terms of reduction of urinary incontinence post intervention. In a study by Colombage et al. [43], using an intra-vaginal pressure biofeedback device, patients learned via video call how to use train their pelvic muscles and how to use the biofeedback device. They were also virtually supervised by a physiotherapist through the 12 weeks course of the study [43]. The study also reports that 81% of the participants had problems when using the intra-vaginal device alone and therefore, it was only used under supervision [43].

Furthermore, Park et al. [44], investigated the effectiveness of digital health in muscular rehabilitation of BC patients after surgery. The study compares the use of augmented reality (AR), which is a combination of the real world and computer-generated content, to standard brochure-based home rehabilitation, in terms of shoulder range of motion, pain, functional results and quality of life. The trial enrolled 50 patients in each group. There was a significant improvement in the aforementioned parameters, however no significant difference between both groups [44].

Dietary interventions are among other digital health services that can potentially serve BC patients. In a trial protocol suspended because of the pandemic that was continued at a later time, Ueland and colleagues [45], enrolled patients with early BC into a group that receives numerous sessions of digital health intervention through video call in comparison to a group that receives a single session. Sessions included interactive nutritional and physical activity interventions. Investigators will study feasibility of the intervention through accrual rate, adherence, retention and accessibility [45].

Since the pandemic, some centers in Argentina started using telemedicine with the intention to improve screening outcomes. Indeed, Malek Pascha et al. [46] estimate that tele-mammography allows the detection of 39 out of 100 new BC cases whereas conventional mammography detects 31 of 100. Cost-effectiveness was also among the advantages, especially for underserved areas [46]. Wearable sensors can also contribute to the early detection of BC at a distance. Elsheakh and colleagues [47] report the use of a textile smart bra that uses microwave settings and reports via an antenna the differences in frequencies sent and received at the level of the breasts. Signals are then analyzed by machine learning algorithms to differentiate benign and malignant tumors. Simulation of the mechanism show a high accuracy rate [47].

In addition, an Australian group developed Finding My Way-Advanced (FMW-A). This is a self-guided psychosocial program that is web-based. Beatty et al. [48], published a study protocol to test its efficacy in improving the quality of life of women with metastatic BC. The protocol compares two telehealth interventions FMW-A and the Australian BC Network’s online application “My Journey”, which is a minimal intervention attention control. The hypothesis is that FMW-A can reduce health system burden while mitigating symptoms and enhancing the quality of life of patients with breast metastatic disease [48]. Examples of on-going trials on telemedicine in BC management after the pandemic are mentioned in Table 2.

**Table 2:** Examples of on-going trials on telemedicine in BC management after the pandemic

**Author, year**	**Country**	**Recruitment**	**Primary outcome**	**Tool**	**Standard**
Henkin et al. [39], 2023	Brazil	Social media, newspapers, and clinics	Cancer-related fatigue	Video call, interactive (MTHE)	HE through folder transfer
Silva Ferreira et al. [40], 2022	Brazil	In person at study site	Sexual function at 6 weeks and 12 weeks after intervention	Sexual function education through audio calls by nurses	No planned intervention
Gordon et al. [41], 2023	US	Institutional records, social media	Feasibility and accessibility	Smart speaker providing supportive care	Tablet platform for supportive care
Elsheakh et al. [47], 2023	Egypt	N/A	Continuous breast monitoring for malignancy	Smart bra with antenna sensor	Standard Screening
Beatty et al. [48], 2022	Australia	In person at study site and through records	Mental quality of life	Multidisciplinary interactive online program	Selected online resources

N/A: not available

PubMed, Embase, the Cochrane Central Register of Controlled Trials were searched for English and non-English reports of studies that investigated telemedicine. Databases were searched until the February 4, 2023. PubMed search terms were: [“Telemedicine” (Mesh) AND “Breast Neoplasms” (Mesh) AND “COVID-19” (Mesh)]. Two reviewers (JZ and ER) independently reviewed the title and abstract of each article to check for eligibility and eliminate duplicates. The reference lists from retrieved articles and the references included in prior relevant systematic reviews and meta-analyses were also checked to ensure that all studies matching the established criteria were included. When the result of a single study was reported in > 1 publication, only the most recent and complete data were included.

Telemedicine offers timely access to care, independent from distance, in a convenient way, limiting the risk of infection, limiting the burden on physical infrastructure, and reducing infectious risk. The aforementioned reasons led to its surge during the COVID-19 pandemic [49].

The acceptability of telemedicine among BC patients is also depended on the phase relative to primary treatment. Indeed, in the primary treatment phase and directly after, patients preferred full physical exam to telemedicine due to fear of recurrence [50]. Although most of responders in their study were satisfied with E-TFU, Merz et al. [33] report that only 43.8% of BC survivors would like to have E-TFU after the pandemic. Oncologists report challenges in the post-pandemic application of telemedicine such as inaccessibility to technologies, lack of infrastructure in clinics for care at a distance, and the problem of cost coverage [26]. A study from the US showed that BC patients coming from households with more than a 150,000 USD income per year were 2.38 times more likely to use telemedicine than those from households with less than 50,000 USD per year. Furthermore, self-insured patients were 70% less likely to adopt virtual appointments in comparison with patients in other insurances [51]. Efforts should be put in making telemedicine more accessible to low-income BC patients, especially that they find it beneficial and easily accessible [52].

Another barrier that telemedicine should overcome is disparities. Indeed, with the ageing of the population, telemedicine should also adapt itself to older women with hearing and vision loss. The use of large computer screens instead of smartphones for a better perception of body language, as well as the help of patients support system for oral communication, are potential solutions for adults with sensory difficulties [50]. Racial inequalities were also reported, where Asian and non-Hispanic white women were more likely to use telemedicine than Black and Hispanic women [53]. More studied should be done to address the way to increase the participation of minorities in telehealth and its accessibility to everyone.

Threats to the applicability of telemedicine in the future also include lack of training of staff and patients, privacy concerns, technical problems and the inability to perform a complete examination [34]. It is also fundamental that teleconsultations receive a high-level medical validation of both their quality and adequacy before their implementation in clinical practice [36]. The legal framework of telemedicine is also an important factor to be considered. From licensing providers to their liability insurance and their prescription of controlled substance, offering medical assistance at a distance remains a challenging task, despite international efforts to facilitate it [54]. The challenges facing telemedicine in BC management after the pandemic have been summarized in Table 3.

**Table 3:** The challenges facing telemedicine in BC management after the pandemic

**Challenges**	**Most concerned groups**
Digital accessibility [26, 50, 53]	Older patients Ethnic groups Patients with sensory disabilities
Infrastructure [26]	Remote regions Developing countries
Staff training and licensing [36]	Small remote centers Developing countries
Reimbursement [51, 54]	Low-income households States with no telehealth regulations

There are also improvements that can be implemented in the video teleconsultation for a better connection between the patients and their physician. Finding a quiet place with adequate lighting and a stable connection is a prerequisite from the physician side. It is also important to have the webcam as close as possible to the patient’s face to establish eye contact while showing the whole upper-body of the physician. Exaggerated facial expressions are also encouraged alongside body gestures that match the patient’ affective state [55].

Future perspectives in telemedicine include the use and development in devices, sensors, and medical applications. For example, smart bras allow the detection of BC for patients from their homes [47]. Additionally, intra-vaginal pressure biofeedback device is another helpful device since it allows the assessment of pelvic functionality in patients. However, without proper live video guidance, many patients encountered difficulties using these methods [43]. This highlights the importance of combining home devices to proper professional support.

## Conclusions

In conclusion, the use of telemedicine during the COVID-19 pandemic was rather beneficial to BC patients and convenient to practitioners. Teleoncology has benefited from the pandemic, not only in order to test new treatment strategies, but also to develop new tools. Since BC survivors, as well as their care providers had positive experiences with teleoncology, it has led to a wider interest in the matter and more upcoming clinical trials. Whether telemedicine will be widely used after the pandemic on larger scales, depend on solving some problems related to its accessibility, reimbursement, and further tools development.

## Abbreviations

AMIE: Addressing Metastatic Individuals Everyday
BC: breast cancer
COVID-19: coronavirus disease 2019
E-TFU: electronic medical record-assisted telephone follow-up
FMW-A: Finding My Way-Advanced
HE: health education
MTHE: multicomponent training and health education
US: United States

## Declarations

### Copyright

© The Author(s) 2023.
